# COVID-19 changing pandemic pattern: why sub-Sahara Africa should reassess the current response approach

**DOI:** 10.11604/pamj.supp.2020.35.2.24921

**Published:** 2020-07-14

**Authors:** Olorunfemi Akinbode Ogundele

**Affiliations:** 1 Department of Community Medicine, University of Medical Sciences, Ondo City, Ondo State, Nigeria

**Keywords:** Approach, community, COVID-19, pandemic, response, sub-Sahara Africa

## To the Editors of the Pan African Medical Journal

As of July 10th 2020, Africa had recorded 541,924 confirmed cases with 12,462 deaths [1]. However, it took nearly 100 days for Africa to reach an initial 100,000 cases, and only 18 days for that number to double to 200,000 [2]. It doubled again to 400,000 cases over the next 20 days [3] raising new concerns among experts across the continent. With many of the health systems poorly funded, inadequately equipped, and poorly staffed with well-trained healthcare workers there are growing fear about the capability of African countries to keep the pandemic under control and the possible huge economic problems that will occur. Africa´s healthcare system is already overburdened; an overwhelming disease outbreak from COVID-19 will spell doom for the continent. The African situation is unique compared to Europe, America or Asia. This is due to the different population structure, a pre-pandemic weak health system that is already stretched in capacity in trying to cope with the double burden of disease with the high prevalence of diseases such as malaria, tuberculosis, HIV/AIDS, and Non-communicable diseases. It may, therefore, be very crucial to look at the Africa context and strategic approach from a different viewpoint than those from other regions of the world as the pandemic is beginning to assume an alarming state in the continent. This is because the approach in other areas of the world might not suffix to attend to the peculiarity of the sub-Sahara Africa countries with the changing pandemic pattern. The obvious, rapidly changing spread pattern over the past few months of the pandemic in the region calls for a review of the approach to response and possible local adaptation taking into consideration the disadvantaged situation of most sub-Sahara African countries.

It is pertinent to realize also that the continent young population makes it different from other parts of the world that are also currently experiencing the pandemic; so does the economic situation. Although Africa´s young population with a median age of 19.7 years [4] was considered a possible protective factor at the beginning of the pandemic, the economic disadvantage might negate this due to the harsh economic realities that make the affordability of even necessary preventive materials difficult and the need to survive economically. The strong communal and religious structure in Africa setting has been a challenge to social and physical distancing; there have been persistent clamours in Nigeria, for example, for reopening of religious and worship centres. The months ahead in this pandemic may be more challenging as more sub-Sahara Africa countries might experience similar uproars due to the strong social cohesion that has been eroded by the pandemic for a while now. Some the Nations have enacted laws and regulations to curb such social and religious gathering; however, the laws will be put to test by the economic and social realities of the continent.

From the points expressed earlier, there is a necessity to reassess the strategic approach employed in the control of the pandemic as countries and nations within the continent continue to respond to the pandemic. There is a need for community engagement and social mobilization rather than a centrally controlled approach. While effort is made at ensuring that the control measures are adhered to, there is need to engage a team of local health authorities, community, opinion, and religious leader to drive the containment effort. Currently, religious, youth and social leader are viewing the response effort from afar and are not relating to it. There is no doubt that some form of training will be required for the team; however, their engagement will boost the response as community confidence in the team will be enhanced in the fight against the pandemic. Such a team will strengthen the call for non-pharmaceutical or clinical control measures in the phase of the economic realities of the countries. A robust response to this rapidly changing pandemic pattern in the continent must shift focus from pharmaceutical and clinical measure towards engaging the community, involving the youth and religious leaders while providing strong leadership in health centrally. Containment of spread at the community level will undoubtedly require community leaders to gather community support for the response. The way to go is for the continent to employ cheaper effective social measures in the control of the pandemic while making an effort to strengthen the weak health systems which at this time will be an arduous task as economic implications of the pandemic begin to take its toll on the continent.

## Conclusion

The pandemic pattern is rapidly changing in the continent; unlike how it started, there is a need to reassess the current response approach by focusing more on social measures than ever before. The current phase requires community engagement with strong health leadership.
